# Experiments with Reference to the Development and Regeneration of Tendons

**Published:** 1884-01

**Authors:** 


EXPERIMENTS WITH REFERENCE TO THE DEVELOPEMENT AND REGENERATION OF TENDONS.—There is no one subject of greater practical importance to the veterinarian than the processes of disease and regeneration in the tendons of the horse; yet there is none upon which we really know less. So far as the work of veterinary authors extends we hardly know the histological characteristics of tendons. We boast of our veterinary schools, yet in our whole literature one can not find a series of scientific articles, based upon direct experiment and microscopic study of the diseases of tendons and the effects of treatment upon the diseased tissues. What are the effects of blisters; the actual cautery; that is what takes place in the tendons, not what is the practical result, how do tendons unite; what takes place in tendons when strained, etc?

These are questions which should be, but are not answered by veterinary writers.

In this regard I beg leave to offer the conclusions with which Dr. Beltzow in the *Archiv f. Mikroskopische Anatomie*, vol. 22, part 4, sums up his most interesting article.

"The results of my experiments may be summed up as follows:

1. "The reactive tendencies of the elements of the tendons, in both warm and cold blooded animals, are of so active a nature that healing (reunion) takes place by proliferation of the cells alone without any preceptable vascular participation *when the tendon is incised, or when cut in two and the separated portions are not to distant from each other;* when the ends are widely separated an active participation of the surrounding connective tissue (Zengewehe) and the formation of granulation tissue takes place as well.

2. The reactivity of the tendon-elements consists essentially of cellular proliferation which occurs as a karyokinestic processes.

3. "Karyokinesis takes place in the fixed cells of the cornea upon irritation and under physiological conditions in the tendons of the embryos of the mammalia.

4. "The original fibres of the tendons do not participate in the processes of healing.

5. "The fibres of the newly developed tissue in all probability develop from the cells and grow (or penetrate) between the fibres of the primary tissue.

6. "*Although this newly developed tissue presents no histological characteristics which distinguished it from the old tissues of the tendon, still it posesses the physiological characteristics of cicatricial tissue; consequently a genuine regeneration of the elements of tendons cannot logically be said to take place.*" B.

